# Prolonged Outbreak of Multidrug-Resistant Shigella sonnei Harboring *bla*_CTX-M-27_ in Victoria, Australia

**DOI:** 10.1128/AAC.01518-20

**Published:** 2020-11-17

**Authors:** Danielle J. Ingle, Patiyan Andersson, Mary Valcanis, Jessica Barnden, Anders Gonçalves da Silva, Kristy A. Horan, Torsten Seemann, Marion Easton, Deborah A. Williamson, Norelle L. Sherry, Benjamin P. Howden

**Affiliations:** a Microbiological Diagnostic Unit Public Health Laboratory, Department of Microbiology and Immunology, Peter Doherty Institute for Infection and Immunity, The University of Melbourne, Melbourne, Australia; b Research School of Population Health, Australian National University, Canberra, Australia; c Department of Health and Human Services, Victoria, Australia; d Department of Microbiology, Melbourne Health, Victoria, Australia; e Doherty Applied Microbial Genomics, Department of Microbiology and Immunology, Peter Doherty Institute for Infection and Immunity, The University of Melbourne, Melbourne, Australia

**Keywords:** ESBL, *Shigella*, epidemiology, genomics

## Abstract

In Australia, cases of shigellosis usually occur in returned travelers from regions of shigellosis endemicity or in men who have sex with men. Resistance to multiple antibiotics has significantly increased in Shigella sonnei isolates and represents a significant public health concern. We investigate an outbreak of multidrug-resistant S. sonnei in Victoria, Australia. We undertook whole-genome sequencing of 54 extended-spectrum-beta-lactamase (ESBL)-producing S. sonnei isolates received at the Microbiological Diagnostic Unit Public Health Laboratory between January 2019 and March 2020. The population structure and antimicrobial resistance profiles were identified by genomic analyses, with 73 previously characterized Australian S. sonnei isolates providing context. Epidemiological data, including age and sex of the shigellosis cases, were also collected. There was a significant increase in cases of ESBL S. sonnei from July 2019. Most of the ESBL S. sonnei isolates (65%) fell within a single cluster that was predominantly comprised of male cases that were characterized by the presence of the *bla*_CTX-M-27_ gene conferring resistance to extended-spectrum cephalosporins. These isolates were also multidrug resistant, including resistance to azithromycin and co-trimoxazole and reduced susceptibility to ciprofloxacin. Our data uncovered a prolonged clonal outbreak of ESBL S. sonnei infection that was likely first introduced by returned travelers and has subsequently been circulating locally in Australia. The emergence of a local outbreak of ESBL S. sonnei with a multidrug-resistant profile, including reduced susceptibility to ciprofloxacin, represents a significant public health threat.

## Abstract (precis)

In Australia, cases of shigellosis usually occur in returned travelers from regions of shigellosis endemicity or in men who have sex with men. Resistance to multiple antibiotics has significantly increased in Shigella sonnei isolates and represents a significant public health concern. We investigate an outbreak of multidrug-resistant S. sonnei in Victoria, Australia. We undertook whole-genome sequencing of 54 extended-spectrum-beta-lactamase (ESBL)-producing S. sonnei isolates received at the Microbiological Diagnostic Unit Public Health Laboratory between January 2019 and March 2020.

## INTRODUCTION

*Shigella* species comprise one of the leading causative agents for severe diarrheal disease globally (1, 2). Whereas the burden of disease is disproportionately experienced by children <5 years old in low- and middle-income countries (1), cases of shigellosis are usually associated with either returned travelers or men who have sex with men (MSM) in high-income countries (HICs) (3–5). Endemic shigellosis in men in HICs is often considered a sexually transmitted infection, with several Shigella sonnei and Shigella flexneri lineages associated with outbreaks in MSM (6–8).

A common characteristic of the MSM-associated outbreaks of *Shigella* infection is the prevalence of multidrug resistance (MDR) to critical oral therapeutics; ciprofloxacin is the first-line agent, and azithromycin and co-trimoxazole are second-line agents. Antimicrobial resistance (AMR) to azithromycin and co-trimoxazole is usually mediated by the acquisition of an MDR plasmid (7), whereas resistance to ciprofloxacin, reported in MSM-associated S. sonnei infection, is due to point mutations in quinolone resistance-determining regions (QRDRs) (5). In the presence of resistance to oral agents, the most frequently used treatment option for severe shigellosis is third-generation (extended-spectrum) cephalosporins, such as ceftriaxone or cefotaxime, which are given intravenously (9). Sporadic cases of extended-spectrum-beta-lactamase (ESBL)-producing S. sonnei have been reported, often in association with travel to Asia (6, 10, 11), but they have not been associated with prolonged outbreaks.

We investigated the recent increase in ESBL-resistant S. sonnei isolates reported from late 2019 to early 2020 in the state of Victoria, Australia. We used whole-genome sequence (WGS) data of S. sonnei, combined with epidemiological data, and contextualized these ESBL isolates with previously characterized Australian S. sonnei isolates to demonstrate the emergence of an ESBL-resistant lineage of S. sonnei circulating in men since October 2019.

## RESULTS AND DISCUSSION

**FIG 1:**
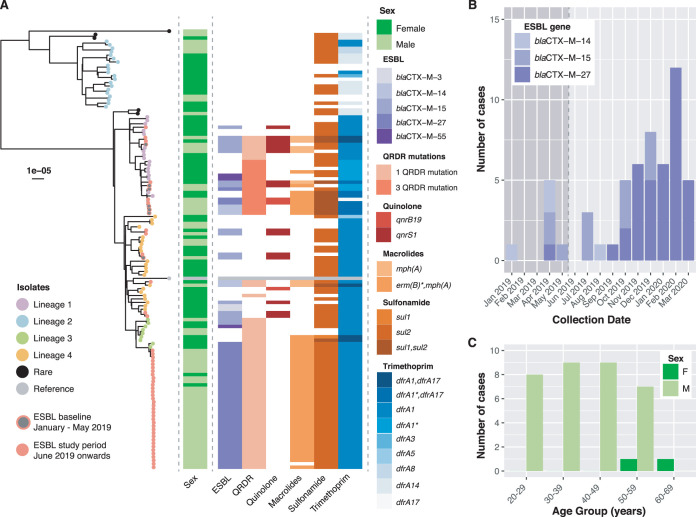
Population structure and antimicrobial resistance profiles of ESBL Shigella sonnei. (A) Midpoint-rooted phylogenetic tree of 54 ESBL Shigella sonnei and 73 contextual isolates. The tips are colored by ESBL status for the novel isolates and by membership to previously established lineages for the contextual isolates. The sex of the patient is shown to the right of the phylogeny. Known genetic determinants for critical antimicrobials are shown as a heatmap. *, partial match (partial gene recovery occurs when 50% to 90% of a protein in the AMRfinder database is covered by a contig at >90% identity). (B) Epidemic curve of ESBL S. sonnei, colored by ESBL gene, received at MDU PHL between 1 January 2019 and 30 March 2020. (C) Patient characteristics of 35 S. sonnei isolates in ESBL outbreak lineage with the histogram stratified by age group and sex.

In total, 54 S. sonnei ESBL isolates were identified in Victoria in the 15 months between January 2019 and March 2020. The inferred population structure illustrated in Fig. 1A shows that the ESBL isolates were distributed within previously defined lineages (4). In the baseline period (January to May 2019), 6 isolates fell in lineage 1 and 1 in lineage 4. Of the 47 novel ESBL isolates received during the study period (June 2019 and March 2020), 35 (74.5%) fell in lineage 3, whereas lineages 1 and 4 each comprised 6 isolates (Fig. 1A). The 35 ESBL lineage 3 isolates formed a genomic cluster, highly suggestive of an outbreak, with a median pairwise distance of 3 single nucleotide polymorphisms (SNPs) (interquartile range, 2 to 4 SNPs). The outbreak isolates and 2 contextual isolates were characterized by the presence of ESBL resistance gene *bla*_CTX-M-27_ accompanied by additional AMR determinants, including *mph*(A) (azithromycin resistance) and *dfrA1* and *sul2* (co-trimoxazole resistance), and decreased susceptibility to ciprofloxacin with a single point mutation in *gyrA* (S83L). Together, these genes confer resistance to the critical oral antibiotics plus extended-spectrum cephalosporins, such as ceftriaxone.

There was a marked increase in ESBL S. sonnei infections in late 2019 and early 2020 compared with early 2019, with 43 (76%) of 54 of cases occurring from October 2019 on (Fig. 1B). The increase was predominately due to isolates carrying *bla*_CTX-M-27_, with both the number and proportion of these isolates increasing over the quarters (Q1 2019, 0/1 [0%]; Q2 2019, 1/6 [17%]; Q3 2019, 1/5 [20%]; Q4 2019 13/19 [70%]; Q1 2020, 23/23 [100%]). All but 3 of 38 *bla*_CTX-M-27_ isolates were part of lineage 3. The remaining 3 isolates with *bla*_CTX-M-27_ fell in lineage 1 and were also characterized by three point mutations in QRDRs. However, the diversity of the AMR profile and demographic characteristics combined with the relatively low incidence of ESBL cases in lineage 1 suggest that these ESBL isolates are likely to be sporadic introductions from different sources. Indeed, the ESBL isolates in lineages 1 and 4 had greater diversity of *bla*_CTX-M_ genes than those in lineage 3, with *bla*_CTX-M-14_ and *bla*_CTX-M-15_ being the more common ESBL mechanisms (Fig. 1A and B).

The population demographics of the cluster of *bla*_CTX-M-27_ genomes in lineage 3 are notably different from those of the sporadic ESBL cases in other lineages and highly indicative of a prolonged outbreak event in Australia. Lineage 3 was previously associated with a high proportion of cases where the identified primary risk factor was MSM (4), and in this study, 33 (94%) of 35 of cases were men (Fig. 1C). The first case in the cluster occurred in September 2019, followed by 2 to 12 cases per month through to the end of the study period. The epidemic curve is highly suggestive of an outbreak event. Furthermore, we note that the AMR profile of these Australian ESBL isolates is consistent with that of a cluster of MDR S. sonnei isolates, with the same ESBL *bla*_CTX-M-27_ gene, that was detected in the United Kingdom between March and November 2018 and identified in a public health alert by Public Health England (PHE) (12). The PHE alert noted that some of the ESBL S. sonnei isolates also clustered with isolates from cases in the United States from male patients who identified as MSM (12). Although investigation of the global prevalence of ESBL S. sonnei isolates was beyond the scope of this study, it does suggest the potential global dissemination of this ESBL sublineage and highlights the need for future public health surveillance to be able rapidly identify and classify high-risk outbreak lineages. Notably, two contextual isolates, which were previously characterized from returned travelers to Southeast Asia (4), had the same AMR profile as the ESBL outbreak cluster. These two isolates were taken from female patients in 2017, which indicates that this sublineage was circulating in Southeast Asia at that time. This suggests that this sublineage of ESBL S. sonnei may have been introduced to Australia by a returned traveler from that region and then transmitted locally.

Here, we report the emergence of a prolonged outbreak of ESBL-resistant S. sonnei in Victoria. This represents a significant public health threat, with members of this prolonged outbreak now resistant to ceftriaxone, co-trimoxazole, and azithromycin and having reduced susceptibility to ciprofloxacin. The latent spread of this ESBL lineage in Victoria has likely occurred in populations with high antimicrobial exposure, coupled with high resistance potential with an existing QRDR mutation, and poses a significant concern for the lineage to become resistant to ciprofloxacin. This may have serious clinical implications, necessitating the use of extremely broad-spectrum antimicrobials, such as carbapenems, and reducing the likelihood of a patient receiving the correct empirical therapy before identification of the MDR *Shigella* strain. Our data also demonstrate the power of enhanced surveillance of enteric pathogens through genomic epidemiology and highlight the need for systematic reporting on ESBL resistance in *Shigella* species, which is not currently required in Australian public health laboratories.

## MATERIALS AND METHODS

Shigellosis is a notifiable disease in Australia. The Microbiological Diagnostic Unit Public Health Laboratory (MDU PHL) is the bacteriology reference laboratory for the State of Victoria (population, ∼6.4 million). MDU PHL receives *Shigella* isolates from primary pathology laboratories for the purpose of further characterization, including phenotypic susceptibility testing and routine WGS. All S. sonnei isolates received by MDU PHL from 1 January 2019 to 31 March 2020 were assessed for ESBL markers (resistance to ceftriaxone and presence of the ESBL gene on WGS). The 54 ESBL-producing isolates identified also had associated epidemiological data, including time of collection and sex and age of the patient. To compare ESBL S. sonnei notifications to those in a previous baseline period, 7 sporadic ESBL S. sonnei isolates received from 1 January 2019 to 30 May 2019 (previously published) were included (5). Details of the ESBL isolates are given in Table S1 in the supplemental material, and short read data are available at BioProject PRJNA319594.

DNA extracts from 47 novel ESBL isolates were prepared using Illumina Nextera XT DNA library chemistry and whole-genome sequenced on a NextSeq500 or NextSeq550. Sequences from 73 Australian S. sonnei isolates broadly representative of the diversity of the previously established population structure were included to provide a contextual framework for the ESBL S. sonnei isolates (4, 5). The 127 genomes were mapped to the reference S. sonnei isolates (GenBank accession no. CP000038) to call SNPs using Snippy v.4.6.0, with filtering of phage regions identified using PHASTER (13), resulting in a core SNP alignment of 4,849 bases. A maximum-likelihood (ML) phylogeny was inferred using IQTree (v.1.6.12) (14) and a GTR+G4 model. The resulting ML phylogeny was midpoint rooted with ape (v.5.3) (15) and phangorn (v.2.5.5) (16) before being visualized with ggtree (v.1.16.6) (17).

*De novo* assembly was performed with SPAdes (v.3.14.0) (18) using the “–isolate” flag. *In silico* determination of known AMR genes in the AMRfinderPlus database was made using abriTAMR (v.2020-01-22.1; https://github.com/MDU-PHL/abritamr). Known point mutations in the QRDRs of *gyrA* and *parC* were identified from Snippy output. Pairwise SNP distances between isolates were determined using harrietR (v.0.2.3; https://github.com/andersgs/harrietr) in R (v.3.6.1).

## Supplementary Material

Supplemental file 1
